# Brainstem Raphe Alterations in TCS: A Biomarker for Depression and Apathy in Parkinson's Disease Patients

**DOI:** 10.3389/fneur.2018.00645

**Published:** 2018-08-07

**Authors:** Daniel Richter, Dirk Woitalla, Siegfried Muhlack, Ralf Gold, Lars Tönges, Christos Krogias

**Affiliations:** ^1^Department of Neurology, St. Josef-Hospital, Ruhr University Bochum, Bochum, Germany; ^2^Department of Neurology, Katholische Kliniken Ruhrhalbinsel, Essen, Germany; ^3^Neurodegeneration Research, Protein Research Unit Ruhr (PURE), Ruhr University Bochum, Bochum, Germany

**Keywords:** sonography, TCS, raphe, apathy, depression

## Abstract

Depression and apathy can both be present in patients with Parkinson's disease (PD) while e. g., essential tremor (ET) patients mostly only report depressive symptoms. In PD, depression has been linked with brainstem raphe (BR) signal alterations in transcranial sonography (TCS) but apathy has not been evaluated in such terms as a putative biomarker. Furthermore, the BR has only been investigated using a singular axial TCS examination plane, although coronal TCS examination allows a much more accurate evaluation of the craniocaudal formation of serotonergic raphe structures in the midbrain area. The objective of this study was to investigate the value of coronal TCS examination for the detection of BR signal alterations and clinically correlate it to apathy in patients with PD, ET and healthy controls (HC). We prospectively included PD patients (*n* = 31), ET patients (*n* = 16), and HC (*n* = 16). All were examined by TCS in the axial and coronal plane with focus on BR signal alterations. LARS and BDI-II scores were conducted to assess apathic and depressive symptoms in the study population. In a detailed analysis we found that the correlation of coronal and axial TCS alterations of BR was very high (rho = 0.950, *p* < 0.001). BR signal alterations were more frequent in PD patients than in ET patients and HC, while it was not different between ET patients and HC. In the PD patient group, BDI-II and LARS scores were negatively correlated to BR signal changes in TCS in a significant manner (BDI-II and axial BR: *p* = 0.019; BDI-II and coronal BR: *p* = 0.011; LARS and axial BR: *p* = 0.017; LARS and coronal BR: *p* = 0.023). Together in this brainstem ultrasound study we find a significant association of BR signal alterations with clinically evident apathy and depression in patients with PD. Therefore, TCS might enable the identification of a subgroup of PD patients which are at higher risk to suffer from or to develop depression or apathy.

## Introduction

Transcranial sonography (TCS) is increasingly applied for the differential diagnosis of Parkinson's disease (PD) patients. Hyperechogenicity of substantia nigra (SN) has been identified as a biomarker and risk factor for PD patients and thus has been included as a diagnostic item in the new MDS research criteria for prodromal PD (1).

Depression and apathy frequently appear in PD patients and can represent early non-motor symptoms (2–4). Several studies have shown that many PD patients are affected by depression, even in the prodromal state of disease (5). Apathy has been described to appear independently from depression (6) and can severely impact quality of life (7). Brainstem raphe (BR) alterations in TCS have been associated with depressed PD patients (8, 9) but were not observed in patients with essential tremor (ET). So far, BR signal alterations have never been examined in relation to apathy and, furthermore, have only been evaluated using a singular axial TCS plane in spite of an additional coronal examination (10).

In this study, we investigated symptoms of apathy and depression by using the Lille apathy rating scale (LARS) and the Beck Depression Inventory II (BDI-II) in PD patients, ET patients and healthy controls. Recently, a new coronal examination plane has been introduced in the field of TCS by our group in order to study Substantia nigra echogogenicity (10). We applied this methodology of an additional coronal TCS examination now for BR alterations and evaluated this finding as a putative biomarker for depression and apathy in PD.

## Materials and methods

### Subjects

We prospectively included patients fulfilling the clinical diagnostic criteria of the UK Parkinson's Disease Society Brain Bank for idiopathic PD (11) or patients meeting the diagnostic criteria for ET (12). Participants with an insufficient transtemporal bone window were excluded. All patients received best medical treatment according to international guidelines for the treatment of advanced Parkinson's disease (13) or essential tremor (14). None of the participants of this study received anti-depressive therapy. Additionally, healthy controls (HC) were included to serve as an age-matched control group. The same clinical assessment and TCS imaging was performed for every subject in this study.

All subjects were included after detailed information about the study and gave written informed consent. In accordance to the Helsinki Declaration of 1975, the study was approved by the local university ethics committee of the Ruhr University Bochum, Germany (approval no. 4961-14).

### Clinical assessment

All PD and ET patients as well as the group of HC underwent clinical neurological assessment, done by a trained and experienced interviewer. Demographic and clinical data was assessed including disease duration and comorbidity (Table 1).

**Table 1:** Demographic, clinical and sonographic data.

	**Parkinson's disease** ** (*n* = 31)**	**Essential tremor** ** (*n* = 16)**	**HC** ** (*n* = 16)**	***p*-value**
Age (years)	69.0 (14.0)	63.5 (21.25)	58.0 (9.25)	0.079^H^
Female (%)/	10 (32.3)/	8 (50)/	10 (62.5)/	0.129^F^
Male (%)	21 (67.7)	8 (50)	6 (37.5)	
Time to first suspected disease	9.0 (6.0)	4.5 (9.75)	–	0.220^U^
Hoehn and Yahr	2.0 (0)	–	–	
MDR-UPDRS III	28.0 (14.0)	7.5 (5.0)	0.5 (2.0)	<**0.001**^H^, ^b^
BDI-II	10.0 (14.0)	12.0 (12.5)	5.0 (8.25)	**0.050**^H^, ^c^
LARS	−24.0 (15.0)	−30.0 (9.0)	−31.0 (4.75)	<**0.001**^H^, ^b^
**TCS**				
Axial SN echogenicity in cm^2^ (Hyperechogen in %)	0.27 (0.10) (90.3)	0.14 (0.04) (12.5)	0.145 (1.0) (18.8)	<**0.001**^H^, ^a^ < **0.001**^F^, ^a^
Reduced BR signal axial (%)	32.3	6.7	6.3	**0.044**^F^, ^a^
Reduced BR signal coronal (%)	29.0	6.7	6.7	0.119^F^

Boldface p-values indicate statistical sgnificance. Values are given in median and (interquartile range). MDR-UPDRS, Part 3 of the Movement Disorder Society Unified Parkinson Disease Rating Scale, BDI-II, Becks Depression Inventory II; LARS, Lille Apathy Rating Scale; TCS, Transcranial sonography.

a Significant difference between the three groups but not between ET patients and HC;

b Significant difference between the three groups and also between ET patients and HC;

c Significant difference between the three groups but not between PD and ET patients;

H Kruskal-Wallis H test;

U Mann-Whitney-U test;

F *Freeman-Halton extension of Fisher's exact test*.

To evaluate motor symptoms of PD, part III of the Unified Parkinson's Disease Rating Scale (UPDRS) (15) was conducted in the entire study cohort. Definition of depression and apathy was based on established scores: using the Beck Depression Inventory II (16) to investigate severity of depression symptoms. A score of ≥19 points was defined as cut-off value for depression (17). Using the Lille Apathy Rating Scale (LARS) (18) to assess apathy, a cut-off-score of ≥-16 was chosen for apathy, since this cut-off-value has shown a high sensitivity of 87% and a high specificity of 93% in diagnosing apathy (18).

### Transcranial sonography

Transcranial sonographic (TCS) examination was performed on the same day as clinical assessment, and was performed by two experienced investigators (DR, CK) being blinded to clinical scores using a phased array ultrasound system equipped with a 2.5-MHz transducer (Aplio® XG Ultrasound System, Toshiba Medicals, Tochigi, Japan). A penetration depth of 150 mm and a dynamic range of 45–50 dB were chosen. Image brightness and time gain compensations were adapted as needed for each examination.

The examination protocol of the axial examination plane was based on previous published recommendations for TCS (19). Using the transtemporal approach, the midbrain and diencephalic examination planes were visualized in axial section. To evaluate SN hyperechogenicity, a planimetric measurement of SN was performed. According to previously published studies (20), we used the mean + 1 SD of SN echogenic area in the group of HC for each plane as upper normal value defining SN hyperechogenicty. In general, an enlarged echogenic area of the SN is a predictive biomarker with high sensitivity and specificity for the diagnosis of PD (10).

**Figure 1:**
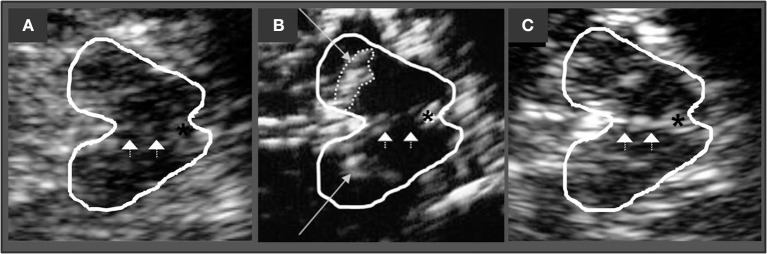
Corresponding mesencephalic axial examination planes. The butterfly-shaped midbrain is outlined for better visualization. The asterisk indicates the aqueduct. Arrowheads indicate the brain stem raphe. **(A)** Raphe structure not visible, grade 0, pathologic finding. **(B)** Echogenic line of the raphe is interrupted, grade 1, pathologic finding. Long arrows indicating the hyperechogenic enlarged area of Substania nigra **(C)** Normal echogenicity, grade 2, normal finding.

The echogenicity of brainstem raphe (BR) was classified semi-quantitatively on a three-point scale: 0 = raphe structure not visible, 1 = slight and interrupted echogenic raphe structure, 2 = normal echogenicity (echogenicity of raphe structure is not interrupted) (Figure 1). Different to the enlarged echogenic area of the SN in PD, a reduced echogenic signal of the BR is thought to visualize changes in the serotonergic system (9).

**Figure 2:**
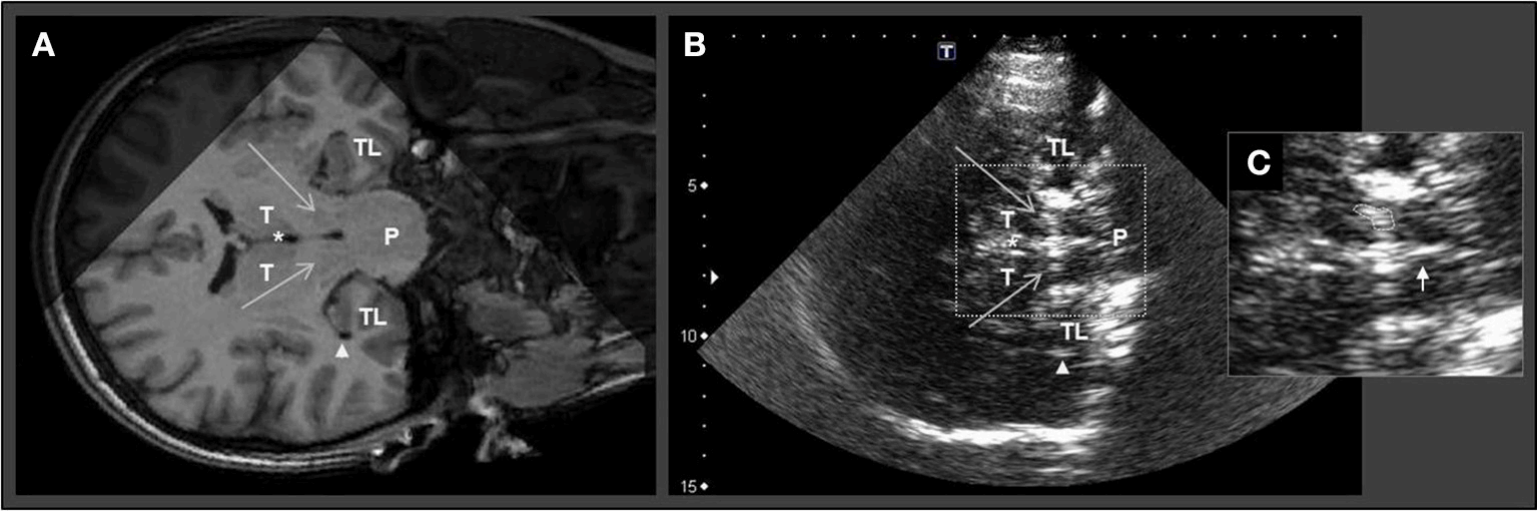
Corresponding MRI and TCS images of coronal examination planes in a patient with idiopathic Parkinson's disease. Coronal MR scanning plane **(A)** and corresponding TCS examination plane **(B)** at brainstem level. **(C)** Zoom in of midbrain structures for planimetric assessment of the echogenic area of substantia nigra and evaluation of brainstem raphe. The large arrows in **(A)** and **(B)** indicate substantia nigra (note that in TCS the SN is displayed echogenic). White asterisks mark the third ventricle. Arrowheads in **(A)** and **(B)** indicate the inferior horn of lateral ventricle. Small arrow in **(C)** indicates the normal appearing brainstem raphe. TL, temporal lobe; P, Pons; T, thalamus.

Similar to the axial examination plane protocol, the temporal bone window was used to assess also the coronal TCS plane: by rotating the transducer for 90°, the hypoechogenic midbrain is visualized in longitudinal view including SN and BR (Figure 2).

The sonographic findings were stored, so that the investigators could perform a second evaluation and classification of the results from the other investigator. In the case of discrepant ratings, a consensus was accomplished, subsequently.

### Statistical analysis

The descriptive statistics are given as median and interquartile range throughout the manuscript. As needed, the range is given additionally. Using appropriate nonparametric tests, the groups were statistically compared and correlation analysis were performed (Kruskal-Wallis H test, Mann-Whitney–U test, Wilcoxon-Test, Fisher's exact test, Freeman-Halton extension of Fisher's exact test, Spearman rho analysis) with SPSS 23.0 for Mac.

## Results

### Study population and clinical features

We screened 36 patients with Parkinson's disease; four patients (11.1%) were excluded owing to an inadequate temporal bone window and one patient (2.7%) was excluded owing to severe dementia. Thus, 31 PD-patients (21 men, 10 women; age = 69.0 [14.0] years) were included into the study. Furthermore, one of the initially 17 patients with essential Tremor was excluded owing to an inadequate temporal bone window resulting in 16 ET patients (8 men, 8 women; age = 63.5 [21.25] years) being included. As control group, 16 healthy subjects were recruited (6 men, 10 women; age = 58.0 [9.25] years). LARS could be performed in 44 participants of the patient population. Detailed information about the clinical characteristics of all participants is demonstrated in Table 1.

#### Symptoms of depression and apathy

Prevalence of depression based on BDI-II definition value differed significantly over the three groups. Owing to the definition, 10 (32.3%) PD patients, 3 (18.8%) ET patients and none of the HC were depressive in our investigation (Freeman-Halton extension of Fisher's exact test, *p* = 0.024). Additionally, there was a strong tendency for difference between PD-patients, ET-patients and HC regarding to the BDI-II Score (Kruskal-Wallis H test, *p* = 0.05). BDI-II scores of PD-patients and ET-patients showed comparable results (Mann-Whitney–U test *p* = 0.770). The BDI-II score was significantly different between PD-patients and HC (Mann-Whitney–U test, *p* = 0.02) while the BDI-II scores of ET-patients and HC did not show a substantial difference as the postulated *p*-value level of statistical significance could not be reached (Mann-Whitney–U test, *p* = 0.056).

Analyzing the appearance of apathy, 12 (38.7%) PD patients, one (7.7%) ET patient and none of the HC were apathic indicating a significant difference in apathy prevalence over the groups (Freeman-Halton extension of Fisher's exact test, *p* = 0.002). Between ET patients and HC no difference in apathy prevalence was found (Fisher's exact test, *p* = 1.000). Regarding to LARS scores, there was also significant difference over the three groups (Kruskal-Wallis H test, *p* < 0.001). This difference in LARS scores appeared significant between PD and ET patients (Mann-Whitney–U test, *p* = 0.023) and significant between ET patients and HC (Mann-Whitney–U test, *p* = 0.045) and PD patients and HC (Mann-Whitney–U test, *p* < 0.001), respectively.

In respect to coexisting apathy and depression, 5 (41.6%) of the apathic PD patients had only apathy and 7 (58.3%) had apathy and depression. Concerning depressive PD patients, 3 (30.0%) had only depression and 7 (70.0%) had both depression and apathy. Interestingly, in these subgroups of PD patients with isolated either depressive (3 patients, 9.7%) or apathic (5 patients, 16.1%) symptoms, only one (3.2%) PD patient with isolated apathy showed an altered BR Signal in TCS.

#### Correlation of LARS and BDI-II

For each group of the study population BDI-II and LARS scores were correlated. Only for PD patients a significant positive correlation was found (PD: rho = 0.657, *p* < 0.001). In ET patients and in the group of HC, no significant correlation was determined (ET: rho = 0.075, *p* = 0.808; HC: rho = 0.131, *p* = 0.630).

#### Transcranial sonography findings

In one ET patient the BR could not sufficiently be evaluated. In one HC only the axial sonographic BR analyzes could be examined. In both cases there was a partially insufficient bone window. Evaluation of BR echogenicity revealed that 10 (32.3%) of the 31 patients with PD, but only one (6.7%) of the remaining 15 patients with ET and only one (6.3%) of the 16 healthy controls exhibited a reduced BR signal in the axial TCS plane indicating structural alterations in this area (Freeman-Halton extension of Fisher's exact test, *p* = 0.044). Using the coronal TCS plane, almost the same distribution of BR alterations in the groups was found (Freeman-Halton extension of Fisher's exact test, *p* = 0.119). Correlation of axial and coronal TCS results in BR region was very high (rho = 0.950, *p* < 0.001) (Table 1).

**Figure 3:**
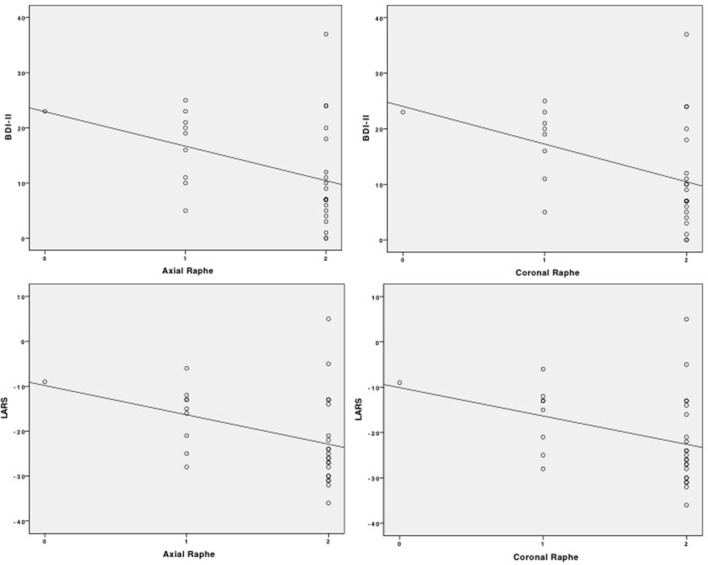
Scatterplots of correlation analysis of BDI-II and LARS with BR signal alteration in the axial and coronal TCS examination.

As BR signal alterations in TCS were very rare in ET patients und HC, correlation analysis of BDI-II, LARS and the sonographic BR results was only performed in the PD group determining a significant negative correlation between BDI-II and BR (rho = −0.420, *p* = 0.019) and LARS and BR (rho = −0.427, *p* = 0.017) using the axial TCS plane. Assessing BR through the coronal TCS plane in the PD population, the negative correlation values between BDI-II and BR (rho = −0.434, *p* = 0.015) and LARS and BR (rho = −0.407, *p* = 0.023) were comparable to the axial TCS assessment. The correlation analysis is also visualized as scatterplots in Figure 3.

Comparing BDI-II and LARS scores between PD patients with and without BR signal alterations in TCS, a significant difference was found. PD patients with BR alterations in axial TCS plane had higher scores in BDI-II (Mann-Whitney–U test, *p* = 0.025) and LARS (Mann-Whitney–U test, *p* = 0.022) compared to PD patients without BR signal changes. The same relation was found for coronal TCS plane by comparing the BDI-II and LARS scores in PD patients and without BR signal changes (BDI-II: Mann-Whitney–U test, *p* = 0.029; LARS: Mann-Whitney–U test, *p* = 0.018). Findings are summarized in Table 2.

Table 2Relation of BR, LARS, and BDI-II.

**Table:** 

**PD patients (*n* = 31)**				
	**BDI-II**		**LARS**	
	**Spearman rho**	***p*****-value**	**Spearman rho**	***p*****-value**
Axial BR evaluation in TCS	−0.420	**0.019**	−0.427	**0.017**
Coronal BR evaluation in TCS	−0.434	**0.011**	−0.407	**0.023**

**Table:** 

	**Axial BR evaluation in TCS**		**Coronal BR evaluation in TCS**		***p*****-value**	
	**BR-**	**BR**+	**BR-**	**BR**+	**Axial**	**Coronal**
BDI-II	19.5 (12.25)	7.0 (11.5)	20.0 (9.5)	7.0 (8.75)	**0.025**^U^	**0.029**^U^
LARS	−14 (10.75)	−26.0 (17.5)	−13.0 (12.5)	−25.5 (14.5)	**0.022**^U^	**0.018**^U^

Boldface p-values indicate statistical significance. Values are given in median and (interquartile range). BDI-II, Becks Depression Inventory II; LARS, Lille Apathy Rating Scale; TCS, Transcranial sonography; BR, Brainstem raphe; BR–, With brainstem raphe alterations; BR+, Without brainstem raphe alterations;

U *Mann-Whitney-U test*.

No differences in BDI-II scores were found if depressive PD patients with and without BR signal alteration in TCS were compared irrespective of applying the axial (Kruskal Wallis H Test, *p* = 0.469) or coronal TCS plane (Mann-Whitney–U test, *p* = 0.257). Moreover, no differences in LARS scores were found, comparing apathic PD patients with and without BR signal alteration in TCS, neither for the axial (Kruskal Wallis H Test, *p* = 0.683) nor for the coronal TCS plane (Mann-Whitney–U test, *p* = 0.792).

Concerning SN hyperechogenicity, the mean ± 1 SD in the group of HC was calculated as 0.19 cm^2^ for axial TCS plane resulting in a robust cut-off value for SN hyperechogenicity of unilateral echogenic SN area of at least 0.20 cm^2^.

28 PD patients (90.3%)*, three* HC (18.8%) and two ET patients (12.5%) showed SN hyperechogenicity (Freemann-Halton extension of Fisher's exact test, *p* < 0.001). Relating to the appearance of axial SN hyperechogenicity in the ET patients compared with the HC, no difference between these groups was found (Fisher's exact test, *p* = 1.000).

In the group of PD patients, the area of the SN in TCS was significantly larger than the SN area of the ET patients and HC (Kruskal-Wallis H test, *p* < 0.001), respectively. No difference in SN areas was found between ET patients and HC (Mann-Whitney–U test, *p* = 1.000).

## Discussion

To our knowledge, this is the first study to correlate BR signal alterations with symptoms of apathy in PD patients. Furthermore, this is the first study which detects BR echogenicity additionally in the recently introduced coronal TCS examination plane. We could show, that BR signal alterations detected by axial TCS examination plane appear more frequently in PD patients compared to ET patients and HC. Similarly, concerning the coronal TCS examination plane, we could confirm a higher prevalence of BR signal alterations in PD patients compared to ET patients and HC. With regard to BR signal changes, the correlation of axial and coronal TCS examination planes was very high indicating no superiority of one TCS plane compared to the other. However, as sonographic raphe analysis is based on a three-point scale we did not expect a substantial difference between the results of both TCS planes a priori.

In other studies, there have been discrepancies in the prevalence of apathy and its status as an independent symptom of PD. Brok et al. found that about 40% of PD patients are suffering from apathy (21). In our study, we found comparable results by determining apathy in about 38.7% of our PD patient population. We could show that a higher grade of apathy was negatively correlated to BR signal alterations in TCS and that PD patients with BR signal changes in TCS had higher scores in LARS. BR alterations in TCS might appear as a structural correlate to apathic symptoms and thus could serve as a correlating biomarker for apathy in PD. Furthermore, TCS might allow to identify PD patients which are at high risk of developing apathy. To validate these findings, a prospective longitudinal study paradigm is needed.

One of the ET patients and none of the HC were apathic according to the definition. Apathy has been occasionally described to occur independently from depression in ET patients (22). In our study, ET patients had higher overall scores in LARS compared to HC indicating a difference in the magnitude of apathic symptoms between these two groups.

In our study, PD patients with BR signal alterations showed significantly higher scores in BDI-II compared to those without BR signal alterations. Independently of the applied TCS plane, the BR signal alteration in TCS was significantly negatively correlated to the BDI-II scores in the PD patients group. This suggests that a structural change in the BR could be associated with the occurrence of depression as well as the severity of depressive symptoms in PD patients and thus might also have a value as a biomarker for depression in PD. Therefore, BR evaluation by TCS might allow to identify a subpopulation of PD patients which are at higher risk to develop a depressive symptomatology and would benefit from a more frequent follow up or more intensive medical treatment. Additionally, we have found a correlation of BR signal changes and apathic symptoms indicating that BR alterations in PD could serve as a biomarker for both apathy and depression. In most of the PD patients apathy and depression were present simultaneously and BDI-II and LARS scores were significantly positively correlated indicating a coexistence of apathic and depressive symptoms in PD.

A reduced BR signal, seen as absent or interrupted echogenic midline structure, is thought to reflect alterations in the serotonergic system and has been reported to be associated with depression in PD patients and with other extrapyramidal neuropsychiatric disorders like Huntington's disease (8, 9, 23). Anatomically, the echogenic midline in general represents various nuclei and fiber tracts of which the dorsal raphe nucleus is one of the main structures. The raphe nucleus is the major origin for serotonin release in the brain (24). A reduced echogenic signal of the BR could be due to alterations in the micro-architecture of this region (25, 26) and thus reflect a serotonin deficiency which is involved in depression pathophysiology.

The pathophysiological basis of apathy is still unknown. Concerning PD, this study found a correlation between sonographic BR alteration and apathy. As the echogenic raphe midline has much more fine anatomical structures than the raphe nuclei, it is also possible that microanatomical changes in other brain regions involved in the serotonin metabolism exist in addition. To our knowledge, sonographic BR signal alterations in PD have only been associated to mood disorders. Interestingly, a recent study has found a significant correlation between tremor severity in PD and raphe dysfunction measured by 123 ioflupane-fluoropropyl-carbomethoxy-3-beta-4-iodophenyltropane single photon emission computed tomography images (27). Further studies should check if sonographic BR signal changes are also linked to motor symptoms in PD.

Because the current BR evaluation in TCS is limited to a three-point scale, the discrimination of BR assessment might not be precise enough to detect differences in the severity of depression. We could not correlate the severity of depression in depressive PD patients to the extent of BR alterations in our study.

Interestingly, no difference with regard to BR signal alterations was found between ET patients and HC, although depression was present in some cases and BDI-II scores were higher in ET patients compared to HC as has been shown before (28, 29). This implies that depression in ET in contrast to PD does not seem to be associated with BR alterations in TCS.

In conclusion, BR signal alterations were found more frequently in PD patients compared to ET patients and HC independent of the applied axial or coronal TCS examination plane. BDI-II and LARS scores of PD patients could be correlated to BR signal alterations of both TCS planes. This indicates that a serotonergic signal disturbance might exist for both depression and apathy and that pharmacologic therapy should take these findings into account. In the case of depression, it has been already reported that a reduced BR signal might be associated with better treatment responses to serotonin-reuptake-inhibitors (SSRI) (30). Further prospective and longitudinal studies are urgently needed to validate these study results in PD in order to substantiate this novel tool to more precisely identify PD and ET patients at risk to develop depression and apathy.

## Author contributions

DR contributed to conceptualizing, organizing the project, analyzing, acquiring, and interpreting the data and writing of the first draft. DW contributed to conceptualizing and revising the manuscript. SM and RG contributed to conceptualizing, organizing the project and revising the manuscript. LT contributed to organizing the project, analyzing, acquiring and interpreting the data and revising the manuscript. CK contributed to conceptualizing, organizing the project, analyzing, acquiring and interpreting the data and revising the manuscript.

### Conflict of interest statement

RG has received consultation fees and speaker's honoraria from BayerSchering, BiogenIdec, MerckSerono, Novartis, Sanofi-Aventis and TEVA. He also acknowledges grant support from BayerSchering, BiogenIdec, MerckSerono, Sanofi-Aventis and TEVA, none related to this manuscript. LT served as a member of AbbVieParkinson Advisory Board. CK received Honoraria for oral presentations or travel grants for scientific meetings from Bayer Vital, Bristol-Meyer Squidd, and Boehringer Ingelheim, none related to this manuscript. The remaining authors declare that the research was conducted in the absence of any commercial or financial relationships that could be construed as a potential conflict of interest.
